# The Effect of Biologics in the Treatment of Multisystem Inflammatory Syndrome in Children (Mis-C): A Single-Center Propensity-Score-Matched Study †

**DOI:** 10.3390/children10061045

**Published:** 2023-06-11

**Authors:** Ozge Basaran, Ezgi Deniz Batu, Ummusen Kaya Akca, Erdal Atalay, Muserref Kasap Cuceoglu, Seher Sener, Zeynep Balık, Erdem Karabulut, Selman Kesici, Tevfik Karagoz, Yasemin Ozsurekci, Yelda Bilginer, Ali Bulent Cengiz, Seza Ozen

**Affiliations:** 1 Department of Pediatric Rheumatology, Hacettepe University Faculty of Medicine, Ankara 06230, Turkey; ozgebasaran@hacettepe.edu.tr (O.B.); ezgideniz.batu@hacettepe.edu.tr (E.D.B.);; 2 Department of Biostatistics, Hacettepe University Faculty of Medicine, Ankara 06230, Turkey; ekarabul@hacettepe.edu.tr; 3 Pediatric Intensive Care Medicine, Life Support Center, Hacettepe University Faculty of Medicine, Ankara 06230, Turkey; selman.kesici@hacettepe.edu.tr; 4 Department of Pediatric Cardiology, Hacettepe University Faculty of Medicine, Ankara 06230, Turkey; 5 Department of Pediatric Infectious Diseases, Hacettepe University Faculty of Medicine, Ankara 06230, Turkey

**Keywords:** multisystem inflammatory syndrome in children (MIS-C), children, comparison, management, biologic, outcomes

## Abstract

Multisystem inflammatory syndrome in children (MIS-C) is a serious condition characterized by excessive inflammation that can arise as a complication of SARS-CoV-2 infection in children. While our understanding of COVID-19 and MIS-C has been advancing, there is still uncertainty regarding the optimal treatment for MIS-C. In this study, we aimed to compare the clinical and laboratory outcomes of MIS-C patients treated with IVIG plus corticosteroids (CS) to those treated with IVIG plus CS and an additional biologic drug. We used the propensity score (PS)-matching method to assess the relationships between initial treatment and outcomes. The primary outcome was a left ventricular ejection fraction of less than 55% on day 2 or beyond and/or the requirement of inotrope support on day 2 or beyond. We included 79 MIS-C patients (median age 8.51 years, 33 boys) followed in our center. Among them, 50 children (25 in each group) were allocated to the PS-matched cohort sample. The primary outcome was observed in none of the patients in the IVIG and CS group, while it occurred in eight patients in the IVIG plus CS and biologic group (*p* = 0.04). MIS-C is a disorder that may progress rapidly and calls for extensive care. For definitive recommendations, further studies, including randomized control trials, are required.

## 1. Introduction

Multisystem inflammatory syndrome in children (MIS-C) is a condition characterized by post-infectious inflammation that occurs as a complication of coronavirus disease 2019 (COVID-19). It was initially identified in childhood during the spring of 2020 [1,2]. Fever, abdominal pain, lymphopenia, and cardiac failure are the symptoms of this new disorder, and it shares several clinical traits with Kawasaki disease (KD), such as conjunctival injection, oral erythema, and cutaneous rash [3,4]. A growing body of evidence supports the idea that this is a post-infectious process that occurs 4–6 weeks after COVID-19.

It can be challenging to distinguish MIS-C from COVID-19 and other hyperinflammatory disorders, including KD and toxic shock syndrome (TSS), and prompt diagnosis is crucial for appropriate care. As a result, studies have been published which created scores for differentiating MIS-C and KD [5,6]. According to the relevant literature, elder age (>5 years), gastrointestinal (GI) system involvement, headache, pericardial effusion, elevated levels of d-dimer and C-reactive protein (CRP), low platelet count, and absence of rash were the main predictors of MIS-C. According to another study, children with MIS-C exhibited elevated liver enzymes and decreased platelet counts. Additionally, these children were more likely to experience GIS involvement, cervical lymphadenopathy, and pulmonary involvement [7]. 

When anti-inflammatory therapies are started promptly in pediatric hyperinflammatory syndromes, like KD or macrophage activation syndrome (MAS), the hyperinflammatory state is frequently reversed, preventing or at least lessening organ damage. To suppress cytokine release and restore immunological homeostasis, therapeutic immune modulation is therefore a cornerstone of the therapy of pediatric inflammatory multisystem syndrome, which is also a cytokine storm syndrome [8,9].

MIS-C is a severe condition that might need treatment in the intensive care unit (ICU). Since it is a critical multisystem disorder, rapid and intense treatment options should be considered. Suppressing systemic inflammation and improving cardiac functions are the main objectives of the therapy [10].

Intravenous immunoglobulin (IVIG) has emerged as the primary treatment for MIS-C due to the similarities of MIS-C and KD and the existence of severe inflammation. However, the distinct characteristics of MIS-C, such as its resemblance to KD shock syndrome and MAS, differentiate it from classical KD. Consequently, IVIG alone may not be sufficient for all patients, leading to the utilization of various immunosuppressive and immunomodulatory agents, including corticosteroids and biologics like interleukin (IL)-1 inhibitors (such as anakinra) and IL-6 blockers (such as tocilizumab) [10,11,12]. Despite these treatment options, the optimal management of MIS-C remains unknown [13]. Hospitalized patients have received combinations of glucocorticoids, IVIG, and biologics with additional immunomodulatory medications. However, the lack of randomized controlled studies has resulted in controversy regarding the optimal treatment approach [14]. 

In this retrospective propensity-score-matched cohort study, our aim was to compare the clinical and laboratory outcomes of MIS-C patients who were given a combination of IVIG plus corticosteroids (CS) versus those who received IVIG plus CS along with an additional biologic drug. Our objective was to assess different treatment strategies according to a defined outcome (not desired outcome) of: a left ventricular ejection fraction of less than 55% on day 2 or beyond and/or the need for inotrope support on day 2 or beyond.

## 2. Materials and Methods

### 2.1. Patients and Data Collection

Patients under the age of 21 who satisfied the case definitions of MIS-C provided by the World Health Organization (WHO) [15] or the Centers for Disease Control and Prevention (CDC) [16] were identified between June 2020 and October 2021. In this period, 79 MIS-C patients were followed up at our center. The WHO and CDC have established the following six primary factors as the basis for the case definition of MIS-C, which include clinical characteristics and the findings of laboratory examinations: (a) pediatric age, (b) persistent fever, (c) high inflammatory biomarkers, (d) organ dysfunction symptoms or signs, (e) absence of a more likely alternative diagnosis, and (f) evidence of COVID-19 infection or SARS-CoV-2 exposure. Patients having any comorbidities or having additional medications were excluded.

We used the propensity score (PS)-matching method to evaluate the relationships between initial treatment and outcomes [17]. Age, gender, comorbidities, the length of the fever at admission, and the COVID-19 PCR results were used to construct the PS. According to the immunomodulatory therapies they received in the first 24 h after admission, we divided the patients into two groups: IVIG plus CS vs. IVIG plus CS and a biologic drug. Fifty children who received either IVIG plus CS (*n* = 25) or IVIG plus CS plus biologic drug (*n* = 25) remained in the matched cohort sample after PS matching.

A thorough assessment of medical records was conducted to gather data on patient demographics, disease characteristics, medications, and medical and family histories. 

The study was done in accordance with the guidelines of the Declaration of Helsinki and received approval from the local ethics committee of our institution with the reference number GO 22/1017 and date 18 October 2022. 

### 2.2. Outcomes

The primary outcome was cardiovascular dysfunction and/or the requirement for an inotrope at least 48 h after starting initial therapy. Cardiovascular dysfunction was defined as a left ventricular ejection fraction of less than 55%. The secondary outcome was the presence of at least one of the following: (1) fever that persists for 48 h after starting therapy, (2) a CRP level that is more than 20 mg/dL 48 h after the start of initial therapy, and (3) second-line therapy, such as additional steroids or IVIG.

### 2.3. Treatment

IVIG was given in a single 12 h infusion at a dose of 2 g/kg. We gave either high-dosage methylprednisolone 10–30 mg/kg/d intravenously for 3 days or 1–2 mg/kg/day oral prednisolone for at least five days or longer. As an anti-IL-1 agent, anakinra was used subcutaneously at a dosage of 2–6 mg/kg/day divided in two doses. As an anti-IL-6 agent, tocilizumab was used as a single IV dose (<30 kg: 12 mg/kg IV; ≥30 kg: 8 mg/kg IV; max.: 800 mg). 

### 2.4. Statistical Analysis

Continuous variables were summarized as mean ± SD for data with a normal distribution and as median (interquartile range) for data with a non-normal distribution. The Kolmogorov–Smirnov test was used to determine if numeric variables had a normal distribution. Independent-sample *t*-tests (for normal distribution data), Mann–Whitney *U* tests (for non-normal distribution data), or repeated measures ANOVA analysis of variance was used to compare parameters in different groups. Categorical variables were given as numbers and percentages and compared using Fisher’s exact test or the chi-square test.

Propensity scores were calculated using a logistic regression model. A one-to-one nearest-neighbor-matching method was applied with a caliper of 0.10, resulting in a comparatively small difference between the matched variable and the control group without replacement. *p* values that were less than 0.05 were considered to be statistically significant. All statistical analyses were performed with the IBM SPSS Statistics version 23.0 statistical software program (IBM Corp., Armonk, NY, USA).

## 3. Results

**Figure 1:**
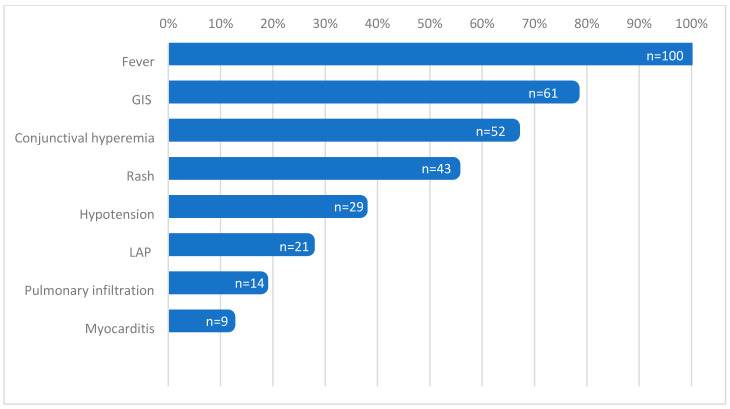
Clinical Symptoms of MIS-C patients in the Whole Cohort. GIS, gastrointestinal system; LAP, lymphadenopathy.

Of the 79 patients (median age 8.51 years; interquartile range 4–12.41 years and 33 boys; 41.7%), 51 (64.5%) received CS plus IVIG plus biologic agent, whereas 28 (35.5%) received only CS and IVIG. A total of 50 patients received anakinra, 1 patient received tocilizumab, and 1 patient received both. While fever was present in all participants, gastrointestinal system involvement, conjunctival hyperemia, and rash were the common presenting features reported in 77.2%, 65.8%, and 54.4% of the patients, respectively. A total of 28 patients (35.4%) received vasoactive/inotropic agents, while 14 patients (17.7%) needed mechanical ventilation support. The median hospital stay was 7 (3–33) days. The clinical features of the whole cohort are summarized in Figure 1.

Patients who received additional biologic therapy were more likely to have a longer median hospital stay (*p* < 0.001) and duration of fever (*p* = 0.02), more hypotension (*p* < 0.001) and pulmonary infiltration (*p* < 0.001), and a greater need for inotropic agents (*p* < 0.001) and O_2_ support (*p* < 0.001) compared with patients who only received IVIG and steroids. All of the patients were discharged in good health and none of the patients died. After PS matching, we found that patients in the IVIG plus CS plus biologic group had significantly longer intensive care unit admission (8 days vs. 1 day, *p* = 0.012), more frequent need for O_2_ supplement, inotrope and plasma exchange therapy (*p* = 0.002, *p* < 0.001, and *p* = 0.005 respectively) than those in the IVIG+CS group (Table 1).

### 3.1. Laboratory Evaluations

The patients who were administered additional biological agents exhibited significantly lower thrombocyte (*p* < 0.001) and lymphocyte counts (*p* = 0.02), decreased levels of albumin (*p* < 0.001), and higher levels of CRP (*p* < 0.001), ferritin (*p* < 0.001), brain natriuretic peptide (BNP) (*p* < 0.001), d-dimer (*p* = 0.02), and troponin (*p* < 0.001) compared with the patients who only received IVIG and steroids. After PS matching, patients in the IVIG plus CS plus biologic group had significantly lower albumin levels (*p* < 0.001), lymphocyte (*p* = 0.002), and thrombocyte counts (*p* = 0.001), and higher IL-6 (*p* = 0.031) levels than those in the IVIG plus CS group. Although not statistically significant, patients in the IVIG plus CS plus biologic group had higher d-dimer, ferritin, and LDH levels and lower Na levels (Table 2). 

### 3.2. Outcomes

While the primary outcome was observed in none of the patients in the IVIG and CS group, it occurred in eight patients in the IVIG+CS and biologic group (*p* = 0.04). Before and after PS matching, there were no significant differences observed between the two groups in terms of the secondary outcomes (Table 3).

## 4. Discussion

The current study analyzes the effect of treatment options on the outcome of patients with MIS-C. To evaluate the primary and secondary outcomes between the steroid plus IVIG versus IVIG plus CS plus biologic groups, the data were analyzed using PS analysis. In this cohort, patients treated with IVIG plus CS and biologic had more intense inflammation and a more severe disease course when compared to those treated with IVIG plus CS. The latter group did not reach the primary outcome criteria, which may reflect that more intense treatment is not required in milder patients. 

The American College of Rheumatology (ACR) has published their comprehensive set of guidelines for MIS-C patients as version 1 in June 2020 and versions 2 and 3 later. In hospitalized patients, they recommended using high-dose IVIG together with low-to-moderate doses of glucocorticoids (1–2 mg/kg/day). Patients with refractory illness should have intensification treatment with high-dose glucocorticoids, anakinra, or infliximab. IVIG, glucocorticoids, and biologic immunomodulators have been the focus of practices and guidelines’ recommendations for second-line therapy [18,19]. Even though there are a comprehensive set of recommendations and protocols, the present therapeutic approaches are purely empirical and not based on evidence. Depending on the treatment facility, therapies have included IVIG, glucocorticoids, and biologic medicines in a variety of combinations. Biologic medications are frequently used effectively as an initial IVIG adjunct treatment, especially in more severe cases. The aforementioned guidelines stress the use of biologics mainly for conditions that are resistant to therapy with IVIG and CS. However, there is currently a lack of evidence evaluating the specific indication, dosing, and timing of anti-cytokine treatments in MIS-C [14,20,21,22]. To prevent a rebound, the ACR consensus guidelines recommended tapering the immunomodulatory drugs over the course of two to three weeks. Depending on this perspective, they hypothesized that including a biologic medication may help maintain a balance between the subacute and acute stages of the illness and act as a steroid-sparing agent [19]. 

Son et al. [23] reported a large cohort study including 518 patients diagnosed with MIS-C. In their propensity-score-matched analysis, they found that IVIG plus steroids were associated with a decreased risk of cardiac function impairment when compared with patients who only had IVIG. They verified that, compared with the IVIG alone group, patients who received initial therapy with IVIG and CS together had better cardiovascular function and a reduced incidence of adjunctive therapy administration. A retrospective cohort study with 181 MIS-C patients revealed a favorable course of fever in the group treated with IVIG and methylprednisolone together as compared with those who received IVIG alone. In these two aforementioned studies, patients in each group received additional treatment or a biologic agent as needed. However, no comparison has been made between these treatments [24]. Since we started IVIG and CS at the same time in our hospitalized MIS-C patients, our cohort did not include patients who received IVIG alone. Therefore, we were unable to assess the efficacy of additional steroids in our cohort, which was one of the limitations of our study.

In 215 patients with MIS-C, Nunez et al. [25] evaluated the short-term results of corticosteroid monotherapy. They compared the clinical and laboratory properties of the patients in three groups: IVIG alone, CS alone, and IVIG plus CS. Compared with the CS group, patients in the IVIG plus CS group had a longer hospital stay. Treatment failure rates did not differ between the CS and IVIG plus CS groups. They found that the disease course was milder in CS patients. Additionally, the occurrence rate of coronary artery involvement and the long-term consequences of the therapies were not evaluated in this cohort of MIS-C patients. Similarly, Licciardi et al. [10] reported 31 patients and compared the outcomes of the patients who either received high- or low-dose methylprednisolone, with a protocol where IVIG infusion was not recommended as the first-line treatment. Eight patients required anakinra step-up therapy because of prolonged fever or elevated CRP, and four patients received IVIG: one for chronic irritability, two for possible CA dilatation, and one for CA involvement. In addition, there were no differences in the overall result between the IVIG alone, steroid alone, and IVIG plus CS therapy groups, according to two large multicenter studies examining 614 and 368 MISC patients [26,27]. Additionally, in the second study, patients who received IVIG and CS had a considerably reduced chance of immunomodulatory therapy intensification than those who only had IVIG therapy. However, neither study assessed the rate of coronary artery involvement in their MIS-C patients or the long-term results of the therapies. Since MIS-C has KD-like aspects and the long-term outcomes of the patients have not been reported, not choosing IVIG in the first-line therapy might be a challenging decision. From that point of view, a recent study reported a single-center experience of cardiac improvement in 40 children with MIS-C. According to their findings, authors suggested that the administration of both IVIG and CS was correlated with a reduced duration of cardiac recovery compared to receiving IVIG alone [28]. On the other hand, physicians need to be aware of fluid overload in case of cardiac failure in critically ill patients. In such cases, division of treatment into two doses can be considered. Moreover, the high cost of IVIG and its limited availability in numerous countries can restrict its utilization as a first-line treatment option. Recently, Channon-Wells et al. published the immunomodulatory treatments of 2009 MIS-C patients in an international cohort study. According to their findings, the study did not observe any significant differences in outcomes between the use of glucocorticoids or intravenous immunoglobulin (IVIG) as single agents, nor between single-agent treatment and combination therapy with IVIG and glucocorticoids as the primary treatments for MIS-C. This suggests that glucocorticoids are equally effective as IVIG or IVIG plus glucocorticoids in the initial management of MIS-C [29]. 

There were no differences in the outcomes between the IVIG alone and IVIG plus CS groups in the studies carried out by Bagri et al. and McArdle et al. [26,27]. In the latter study, the groups did not vary in terms of inflammatory markers or escalation of immunosuppressive therapy. In the present study, both before and after PS matching, patients who received additional biologic therapy exhibited more severe clinical and laboratory features. After the PS matching, patients having combination therapy had a significantly higher primary outcome. Although a similar or better response was expected in patients who received more severe treatment, this result could not be demonstrated in our patients. These results imply that individuals with more serious illnesses may have required further biologic treatments earlier. On the other hand, in secondary outcomes (persistent fever, high CRP levels, and second line therapy after day 2), similar results were obtained in both groups of patients. In addition to these, we showed that the patients who had started the IVIG plus CS plus biologic agent had significantly lower platelet and lymphocyte counts and albumin levels and higher d-dimer, ferritin, and CRP levels. Those laboratory parameters might guide the clinicians to start aggressive treatments in the early phase of the disease.

Patients diagnosed with MIS-C exhibit prominent signs of high inflammation, potentially accompanied by shock and cardiac dysfunction. This condition necessitates a thorough differential diagnosis to distinguish it from other disorders like TSS, KD, KD shock syndrome, or MAS. In fact, MIS-C is considered to be encompassed within the umbrella of systemic inflammatory response syndrome [30]. While the exact mechanism underlying MIS-C remains largely unknown, there is suggestive evidence indicating that IFN-γ and monocytes can play a crucial role in the pathogenesis of this condition [31,32]. Acute MIS-C is marked by increased levels of systemic inflammatory cytokines, such as IL-1β, IL-6, IL-8, IL-10, IL-17, and tumor necrosis factor-α [33]. The signaling of IL-1 receptors has also been associated with the development of both KD and MIS-C, and IL-1 blockers are being used for the treatment of patients with these conditions [34]. Furthermore, a recent study by Zhu et al. suggested that activated neutrophils express IL-1β in patients with MIS-C and KD [11]. With all these results, considering the elevated levels of inflammatory markers and intense inflammatory profile observed in MIS-C patients, the utilization of biologic agents appears to be a reasonable approach. Despite being an area of contention in the literature, the use of biologic agents like anakinra and tocilizumab as the first-line treatment, along with other immunosuppressive drugs, for patients with MIS-C remains controversial. Brisca et al. conducted an observational single-center study involving 23 MIS-C patients [35]. Considering the severity of the patients’ condition upon admission, a treatment protocol comprising several stages was implemented to address the inflammatory response. This involved assessing the extent of cardiac features including echocardiographic findings and cardiac enzyme levels, as well as identifying any abnormalities in blood tests that could suggest MAS. The patients were then treated accordingly based on these assessments. They had included four treatment protocols, one of which involved the administration of IVIG along with CS and anakinra. Of the 23 MIS-C patients, within the first 48 h after admission, 6 patients only received IVIG, 14 patients received IVIG along with high dose intravenous methylprednisolone (2–3 mg/kg/day in 9 patients and pulses of 30 mg/kg/day in 5 patients), and 3 patients had IVIG, methylprednisolone, and anakinra. Importantly, none of the patients in the study required admission to ICU, invasive mechanical ventilation, extracorporeal circulatory or respiratory support, or the administration of inotropic drugs. Based on their favorable observation outcomes, Brisca et al. concluded that prompt identification and addition of anti-inflammatory therapy could be crucial in achieving a favorable disease course and preventing the need for inotropic drugs, mechanical ventilation, or ICU admission [35]. However, it should be noted that the study had a relatively small sample size and only reported short-term outcomes. Furthermore, the researchers did not compare the outcomes, clinical characteristics, or laboratory features between the different treatment groups. In a retrospective cohort study carried out by Chang et al., they analyzed MIS-C cases from a US surveillance registry between November 2020 and December 2021. They aimed to assess the outcomes of patients who took IVIG and CS versus those who received anakinra in combination with IVIG and/or CS within the initial two days of treatment. Among the 1516 MIS-C patients included in the study, 193 (13%) received anakinra alone or in combination with other immunosuppressive drugs as their initial treatment. The authors noted considerable variation in the use of anakinra as an initial treatment for MIS-C. However, contrary to previous literature findings, the study did not find any overall short-term cardiovascular improvement associated with the prompt starting of anakinra to IVIG and/or CS compared to the use of IVIG and CS alone [34]. 

This study has some limitations. First of all, even though we utilized PS matching, this is not a prospective randomized study. Second, since this is a single-site study, our sample size was comparatively small. Moreover, we had fewer numbers after the PS matching. We included only baseline features while constructing the PS since increasing the number of factors severely limited the number of patients in the subgroups. This might have introduced bias to the comparisons between IVIG and biologic groups. In addition, we were unable to assess the effectiveness of steroid-only or IVIG-only treatment modalities since all patients received CS and IVIG at the same time when they were admitted; the dosage and route of administering CS were non-standard; and only two of our patients received tocilizumab. Therefore, due to this limited data, we could not make comparisons between different biologic agents. Finally, we did not compare the long-term effects of utilizing additional biologics since we did not have follow-ups with our patients. The strength of our study is that there are only a few studies discussing the usage of biologic medications in MIS-C patients to date. 

## 5. Conclusions

MIS-C cases have started to decrease; however, this article is important in terms of determining treatment options in patients who present with a secondary hyperinflammatory response secondary to various etiologies. Although we could not show any significant differences in clinical features, our results showed that lower platelet and lymphocyte counts and albumin levels and higher d-dimer, ferritin, and CRP levels might justify the use of aggressive treatments in the early phase of the disease. On the other hand, we need continued research and randomized controlled trials for optimal management and timing of the immunosuppressive agents to achieve a satisfactory clinical outcome without unnecessary aggressive treatments.

## Figures and Tables

**Table 1:** Demographic and Clinical Features of the Patients with Multisystem Inflammatory Syndrome in Children.

	Whole Cohort			Cohort after Propensity Score Matching		
	IVIG+CS (n = 28)	IVIG+CS+Biologic (n = 51)	*p* value	IVIG+CS (n = 25)	IVIG+CS+Biologic (n = 25)	*p* value
Sex (M/F)	11/17	22/29	0.74	15/10	15/10	1
Age at diagnosis mo, (mean)	85.89 (±59.23)	109.56 (±55.32)	0.08	85.44 (±61.14)	93.84 (±49.10)	0.595
Rash, n (%)	12 (42.9%)	31 (60.8%)	0.126	11 (44%)	17 (68%)	0.087
Lymphadenopathy, n (%)	9 (32.1%)	12 (23.5%)	0.407	9 (36%)	8 (32%)	0.76
GIS symptoms, n (%)	20 (71.4%)	41 (80.4%)	0.364	14 (56%)	18 (72%)	0.23
Dyspnea, n (%)	0	9 (17.6%)	**0.01**	0	3 (12%)	0.12
Pulmonary infiltration, n (%)	0	14 (27.5%)	**<0.001**	0	4 (16%)	0.11
Pleural effusion, n (%)	0	11 (21.6%)	**<0.001**	0	4 (16%)	0.11
Hypotension, n (%)	2 (7.1%)	27 (52.9%)	**<0.001**	2 (8%)	11 (44%)	**0.004**
Myocarditis, n (%)	3 (10.7%)	6 (11.7%)	0.88	3 (12%)	2 (8%)	1
Pericardial effusion, n (%)	4 (14.2%)	12 (23.5%)	0.32	4 (16%)	4 (16%)	1
Mechanical ventilation, n (%)	0	14 (27.5%)	**0.002**	0	5 (20%)	**0.02**
LVEF < 55, n (%)	1 (3.6%)	12 (23.5%)	**0.02**	1 (4%)	2 (8%)	0.50
Intensive Care Unit Admission, n (%)	1 (3.6%)	21 (41.2%)	**<0.001**	1 (4%)	8 (32%)	**0.01**
Need for O_2_ supplement, n (%)	0	19 (37.3)	**<0.001**	0	8 (32%)	**0.002**
Need for inotrope, n (%)	1 (3.6%)	27 (52.9%)	**<0.001**	1	12 (48%)	**<0.001**
Need for plasma exchange, n (%)	1 (3.6%)	20 (39.2%)	**<0.001**	1	9 (36%)	**0.005**
Duration of fever, days (median)	4 (1–10)	5 (1–12)	**0.02**	5 (1–10)	5 (0–7)	0.55
Duration of admission, days (median)	5 (3–11)	8 (4–33)	**<0.001**	5 (3–11)	8 (5–27)	**0.002**

M, male; F, female; IVIG, intravenous immune globulin; CS, corticosteroid; GIS, gastrointestinal system; LVEF, left ventricular ejection fraction. Statistically significance is shown in bold.

**Table 2:** Laboratory features of the patients with multisystem inflammatory syndrome in children (MIS-C).

	Whole Cohort			Cohort after Propensity-Score Matching		
	IVIG+CS (n = 28)	IVIG+CS+Biologic (n = 51)	*p* value	IVIG+CS (n = 25)	IVIG+CS+Biologic (n = 25)	*p* value
Hb g/dL (±SD)	12.02 (±1.50)	11.12 (±1.67)	0.48	11.83±	11.66 (±1.40)	0.67
Na mEq/L (136–146) (±SD)	134.5 (±2.11)	135.1 (±4.3)	0.36	134.44 (±2.21)	133.48 (±3.05)	0.21
Albumin (g/dL) (±SD) (3.5–5.2)	3.6 (±0.59)	3.02 (±0.50)	**<0.001**	3.63	3.04	**<0.001**
White blood cell/mm^3^, median	10,200 (3200–27,600)	11,300 (1300–45,000)	0.99	9500 (3200–27,600)	11,300 (1300–29,900)	0.95
Lymphocyte/mm^3^, median	1300 (100–9900)	810 (200–7300)	**0.002**	1300 (100–9900)	710 (280–2700)	**0.002**
Thrombocyte, median	255,000 (62,000–766,000)	155,000 (49,000–520,000)	**<0.001**	260,000 (62,000–766,000)	159,000 (49,000–439,000)	**0.001**
CRP (mg/dL), median	8.8 (1.2–35.59)	19.29 (0.12–35.40)	**0.002**	10.5 (1.2–35.59)	20.0 (0.12–35.11)	**0.02**
ESR (mm/h), median	29.0 (2–88)	29.5 (2–96)	0.74	38 (5–88)	29 (2–96)	0.68
D-dimer (mg/L) median (0–0.55)	2.22 (0.43–35.20)	4.20 (0.7–80.00)	**0.02**	2.24 (0.43–24.39)	3.79 (0.7–56.20)	0.13
Ferritin (ng/mL), median (11–307)	215 (27–1453)	456 (3.6–3875)	**<0.001**	275.6 (27–1453)	407.0 (3.6–2743)	0.06
BNP (pg/mL), median (0–100)	47 (10–1655)	489 (10–5704)	**<0.001**	59.0 (10–1655)	201.0 (15–2287)	**0.02**
LDH (U/L), median (180–430)	292 (133–671)	320 (133–1013)	0.26	292.0 (133–671)	324.0 (203–752)	0.23
IL-6 (pg/mL) (<6.4)	43.5 (3.9–1176)	65.28 (5.93–4752)	0.06	38.0 (3.9–1176)	89.2 (6.1–4752)	**0.031**
Troponin ng/L (8.4–18.3)	3.65 (2.3–5928)	55 (2.3–9248)	**<0.001**	5.2 (2.3–5928)	22.7 (2.3–4492)	0.02

IVIG, intravenous immune globulin; CS, corticosteroid; CRP, c-reactive protein; ESR, eritrocyte sedimentation rate; BNP, brain natriuretic peptide; LDH, lactate dehydrogenase; IL-6, interleukin 6. Statistically significance is shown in bold.

**Table 3:** Outcomes in different treatment groups in patients with multisystem inflammatory syndrome in children (MIS-C).

	Whole Cohort Propensity Score Match			Cohort after Propensity Score Matching		
	IVIG +CS (n = 28)	IVIG+CS+Biologic (n = 51)	*p*	IVIG +CS (n = 25)	IVIG+CS+Biologic (n = 51)	*p*
Primary outcome, n (%)	0	17 (33%)	0.0	0	8 (32%)	**0.04**
Secondary outcome						
Second-line therapy n (%)	2 (8%)	5 (9.8%)	0.69	3 (12%)	8 (32%)	0.08
CRP level > 20 mg/dL n (%)	3 (10.7%)	6 (11.7%)	0.6	3(12%)	2 (8%)	1.0
Persistent or recurrent fever n (%)	2 (8%)	5 (9.8%)	0.69	2 (8%)	2 (8%)	1.0

IVIG, intravenous immune globulin; CS, corticosteroid; CRP, c-reactive protein. Statistically significance is shown in bold.

## Data Availability

The data presented in this study are available on request from the first author.
